# Nitric Oxide and Iron Signaling Cues Have Opposing Effects on Biofilm Development in Pseudomonas aeruginosa

**DOI:** 10.1128/AEM.02175-18

**Published:** 2019-01-23

**Authors:** Xinyi Zhu, Scott A. Rice, Nicolas Barraud

**Affiliations:** a The Singapore Centre for Environmental Life Sciences Engineering, Nanyang Technological University, Singapore; b Interdisciplinary Graduate School, Nanyang Technological University, Singapore; c The School of Biological Sciences, Nanyang Technological University, Singapore; d ithree Institute, University of Technology Sydney, Sydney, New South Wales, Australia; e Genetics of Biofilms Unit, Institut Pasteur, Paris, France; University of Bayreuth

**Keywords:** biofilm dispersal, nitric oxide, *Pseudomonas aeruginosa*, iron

## Abstract

While both iron and nitric oxide (NO) are redox-active environmental signals shown to regulate biofilm development, their interaction and roles in regulating biofilms have not been fully elucidated. In this study, exposure of Pseudomonas aeruginosa biofilms to exogenous NO inhibited the expression of iron acquisition-related genes and the production of the siderophore pyoverdine. Furthermore, supplementation of the culture medium with high levels of iron (100 μM) counteracted NO-induced biofilm dispersal by promoting the rapid attachment of planktonic cells. In the presence of iron, biofilms were found to disperse transiently to NO, while the freshly dispersed cells reattached rapidly within 15 min. This effect was not due to the scavenging of NO by free iron but involved a cellular response induced by iron that led to the elevated production of the exopolysaccharide Psl. Interestingly, most Psl remained on the substratum after treatment with NO, suggesting that dispersal involved changes in the interactions between Psl and P. aeruginosa cells. Taken together, our results suggest that iron and NO regulate biofilm development via different pathways, both of which include the regulation of Psl-mediated attachment. Moreover, the addition of an iron chelator worked synergistically with NO in the dispersal of biofilms.

**IMPORTANCE** Nitric oxide (NO), which induces biofilm dispersal, is a promising strategy for biofilm control in both clinical and industrial contexts. However, competing environmental signals may reduce the efficacy of NO. The results presented here suggest that the presence of iron represents one such environmental cue that antagonizes the activity of NO as a biofilm-dispersing agent. Based on this understanding, we developed a strategy to enhance dispersal by combining NO with an iron-scavenging agent. Overall, this study links two important environmental signals, iron and NO, with their roles in biofilm development and suggests new ways for improving the use of NO in biofilm control strategies.

## Abstract (precis)

Nitric oxide (NO), which induces biofilm dispersal, is a promising strategy for biofilm control in both clinical and industrial contexts. However, competing environmental signals may reduce the efficacy of NO. The results presented here suggest that the presence of iron represents one such environmental cue that antagonizes the activity of NO as a biofilm-dispersing agent. Based on this understanding, we developed a strategy to enhance dispersal by combining NO with an iron-scavenging agent. Overall, this study links two important environmental signals, iron and NO, with their roles in biofilm development and suggests new ways for improving the use of NO in biofilm control strategies.

## INTRODUCTION

Biological life cycle transitions are often regulated by the interplay between genetic elements and chemical or environmental cues (1). An understanding of these interactions may enable a better control of developmental processes. In bacterial biofilms, the opposing stages of attachment and dispersal are both controlled by a number of external cues and a network of specific genes. The attachment of bacterial cells to biotic or abiotic surfaces is mediated by extracellular polymeric substances (EPS) that include adhesive proteins and polysaccharides as well as extracellular DNA (eDNA) (2). The opportunistic pathogen and model biofilm-forming organism Pseudomonas aeruginosa produces three types of exopolysaccharides, namely, alginate, Pel, and Psl, as well as several proteins that have been shown to be involved in biofilm formation. The adhesin CdrA strongly binds Psl and anchors cells to the EPS matrix or, when secreted, cross-links fiber-like Psl strands, thus stiffening the gel-like EPS matrix (3). In P. aeruginosa, alginate, Pel, and Psl are partly regulated by bis-(3′-5′)-cyclic dimeric GMP (c-di-GMP), an intracellular secondary messenger conserved across bacterial species (4, 5). In many bacteria, c-di-GMP levels are controlled by multiple enzymes (phosphodiesterases [PDEs] and diguanylate cyclases [DGCs]), some of which are associated with sensory domains (e.g., PAS domain) capable of responding to extracellular stimuli, including environmental cues (e.g., oxygen and redox conditions, light, and starvation) as well as cell-to-cell signals (4). While high levels of c-di-GMP, via interaction with transcriptional regulators and direct effectors, usually promote attachment, lower intracellular levels downregulate attachment, induce the expression of motility genes, and trigger dispersal.

There are a variety of environmental signals that can induce biofilm dispersal. For example, biofilm dispersal can be triggered by low levels of nitric oxide (NO) (6, 7), oxygen depletion (8, 9), and changes in temperature (10) as well as changes in iron levels and nutrient availability (11–14). Among these cues, NO has attracted particular interest as its role in biofilm dispersal appears to be conserved across bacterial species. Thus, several promising strategies have been developed to deliver NO and disperse antimicrobial-resistant biofilms that could find applications across a range of industrial and clinical settings (15). NO is a hydrophobic molecule and a highly reactive free radical (16). At low nontoxic concentrations (nanomolar range), NO induces biofilm dispersal, while higher concentrations may cause nitrosative damage to bacterial cells. In P. aeruginosa, NO disperses biofilms through the stimulation of phosphodiesterase activity, resulting in decreased intracellular c-di-GMP concentrations and involving the periplasmic protease LapG (7, 17). Several sensors of NO have been identified, including a newly characterized heme-binding sensor protein, NosP, that is involved in regulating biofilm dispersal in P. aeruginosa and is highly conserved among bacteria (18). While the exogenous addition of NO can disperse a significant portion of biofilms, the addition of NO generally does not disperse all of the biofilm (6). We have recently shown that the nondispersing cells become insensitive to NO as a consequence of the production of flavohemoprotein, which scavenges NO (19).

NO can bind to most transition metals (20), of which, iron is one of the best understood. For example, NO binds to heme sensors and affect cytochromes or iron-sulfur clusters (21). Interestingly, iron has been shown to impact biofilm developmental processes, where low or high iron concentrations inhibit or increase biofilm formation, respectively. Thus, iron and NO have opposing activities. However, the direct link between iron and NO in the regulation of biofilms remains poorly understood. Iron is an essential nutrient to sustain bacterial growth, and bacteria have evolved several strategies for iron acquisition and uptake (22), which may be especially important under conditions of high cellular density, such as in biofilms. Mature biofilms exhibit gene expression profiles consistent with iron limitation (23). Previous studies have shown that iron availability controls biofilm formation through several mechanisms, including modulating quorum sensing (QS) cell-cell signaling, stimulating DNA release, and enhancing the production of Psl polysaccharides (13, 24, 25). Generally, under iron-limiting conditions, P. aeruginosa does not form biofilms or only forms flat unstructured biofilms (13, 26). In contrast, under iron-replete conditions, biofilm formation is increased (14, 24). Furthermore, pyoverdine production is reduced in P. aeruginosa cells with lower c-di-GMP levels (27–29). Pyoverdine is a high-affinity siderophore produced by P. aeruginosa to acquire iron in an iron-limiting environment (30–32). The mechanisms regulating these effects remain to be fully elucidated, and to date, no c-di-GMP-dependent receptor involved in *pvd* transcription has been identified. Moreover, P. aeruginosa Δ*pvdA*, Δ*pvdS*, and Δ*fpvA* mutant strains, which are defective in genes important for pyoverdine synthesis, signaling, and uptake (33, 34), were shown to form thin layer biofilms; for the Δ*pvdA* mutant, the biofilm mushroom-like structure was restored when pyoverdine was exogenously added (34). Iron may also affect biofilm formation through the QS signaling pathway. The parental strain forms biofilms poorly under an iron-limiting condition, while the structured mushroom-like biofilm formation was largely restored in the *rhlI* mutant (35). Moreover, a recent study showed that in P. aeruginosa, high iron (50 and 100 μM FeCl_3_) promoted Psl production and induced biofilm formation (24). Psl was also found to bind both ferrous and ferric iron and store iron to further induce Psl-dependent biofilm formation (24).

In this study, transcriptomic analysis of the NO-mediated dispersal response was performed to better understand the physiological changes induced by NO. NO-treated cells had reduced expression of genes for the synthesis of pyoverdine and the lower-affinity siderophore pyochelin (36) as well as other iron acquisition-related genes. Thus, a potential link between iron acquisition and NO-mediated dispersal was further explored. Supplementation of the culture medium with high levels of iron overrode NO-induced biofilm dispersal by promoting the rapid attachment of planktonic cells, which was linked to the production of Psl. In contrast, the dispersal response appeared to involve changes in Psl-mediated attachment of P. aeruginosa cells. Finally, the addition of the iron chelator 2,2′-bipyridine (Bipy) showed a synergetic effect with NO in dispersing biofilms. Simultaneous treatment of biofilms with NO and an iron chelator might enhance biofilm dispersal in environments where high iron levels might inhibit the ability of NO to disperse biofilms.

## RESULTS

### NO inhibits expression of iron acquisition-related genes and pyoverdine production.

To elucidate the molecular pathway of NO-induced dispersal, this study compared transcriptomic profiles of P. aeruginosa untreated, planktonic, and biofilm cells to those of NO-induced dispersed bacteria as well as cells remaining within biofilm structures after treatment with the NO donor, spermine NONOate (SP-NO). The methods of the transcriptomic experiment are described in Text S1 in the supplemental material. The results showed that the expression levels of most iron acquisition-related genes in NO-treated biofilms and dispersed cells were lower than in untreated cells (see Tables S1 and S2). Several extracytoplasmic function sigma factors (ECF-σ) controlled by the ferric uptake regulator Fur (37), including *pvdS* and *femI*, were downregulated in NO-treated cells (dispersed cells and biofilms) compared to that in untreated cells. Genes for pyoverdine synthesis were downregulated, such as *pvdA* (34), whose expression was 7-fold lower in NO-treated biofilms than in untreated biofilms and 6-fold lower in dispersed cells than in planktonic cells. *tonB1*, an essential component of the siderophore-mediated iron uptake system (38), was also decreased by 11-fold in NO-treated biofilms compared to that in untreated biofilms. Iron receptors, including those encoded by *fpvA*, *optI*, and *hasR*, exhibited lower expression levels in NO-treated biofilms than in untreated biofilms. Among these genes, the expression level of *fpvA* was 8-fold lower in NO-treated biofilms than in untreated biofilms. Moreover, the expression of *pvdQ* was nine times lower than in untreated biofilms, and *pchA-D* and *pchR* (39), involved in pyochelin biosynthesis, were also downregulated in NO-treated biofilms. In addition, the expression levels of phenazine biosynthesis genes *phzA1*, *phzB2*, *phzC1*, *phzC2*, *phzD1*, *phzD2*, *phzE1*, *phzE2*, *phzF1*, *phzF2*, *phzG1*, and *phzG2*, which encode redox-active pigments involved in QS, virulence, and iron acquisition (40), were decreased at least 4-fold in NO-treated biofilms. *phzA1*and *phzB1*were downregulated in dispersed cells compared to that in planktonic cells. Therefore, the data suggest that the pyoverdine and pyochelin synthesis genes, as well as iron acquisition-related genes, were generally reduced after NO treatment. In contrast, *bfrB*, encoding a bacterioferritin, which is an important iron storage protein in P. aeruginosa (41), was highly upregulated in NO-treated remaining biofilms (72-fold) and NO-dispersed cells (77-fold) compared to that in untreated cells.

**FIG 1:**
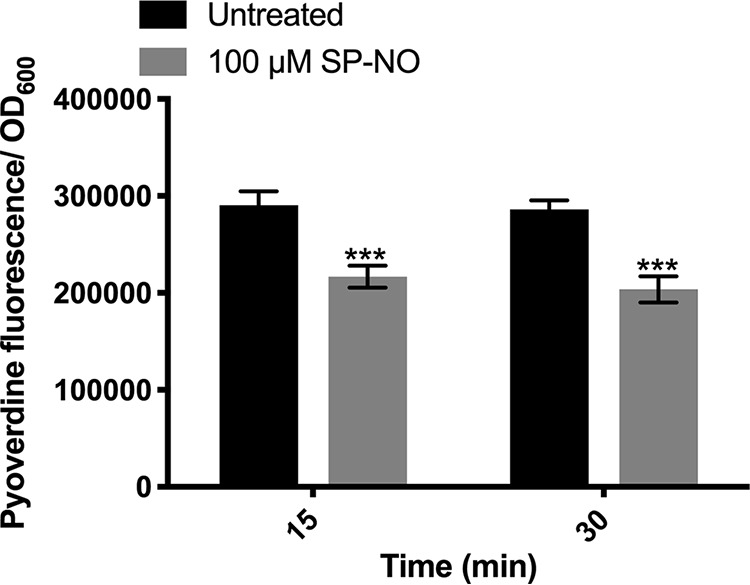
NO inhibits pyoverdine production in P. aeruginosa. Biofilms were grown in multiwell plate batch cultures for 6 h and treated with 100 μM NO donor SP-NO for 15 or 30 min. Relative fluorescence units (RFU) of pyoverdine were calculated by normalizing the pyoverdine fluorescence to the OD_600_ measurement of the culture density. Error bars indicate standard deviations (*n* = 8). ***, *P ≤ *0.001 versus untreated control samples.

Since the expression levels of most iron acquisition-related genes (e.g., *pvdA*, *pvdS*, and *fpvA*) were downregulated after exposure to 100 μM NO donor SP-NO, the effect of NO on pyoverdine production was further investigated. Pyoverdine production was reduced 25% after adding 100 μM SP-NO for 15 min (Fig. 1). Similar results were observed after 30 min of exposure. Overall, these data suggest a link between the repression of iron acquisition-related genes induced by NO and the regulation of biofilm dispersal.

### Iron overrides NO-induced dispersal independent of NO-scavenging pathways.

**FIG 2:**
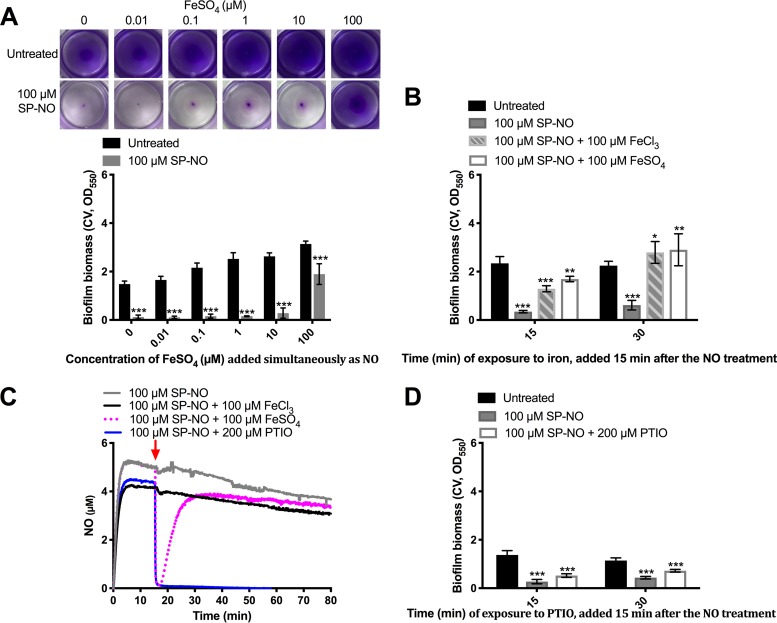
Iron overrides NO-induced biofilm dispersal. (A) P. aeruginosa biofilms were grown in multiwell plate batch cultures for 6 h and treated with 100 μM NO donor SP-NO and different concentrations of FeSO_4_ simultaneously for 30 min before quantifying the biofilm biomass by CV staining. Each image represents the stained biofilms. Biofilms grown in multiwell plate batch cultures for 6 h and treated with 100 μM SP-NO for 15 min were subsequently treated with 100 μM FeCl_3_ or FeSO_4_ (B) or 200 μM PTIO (D) for 15 or 30 min before CV staining. Error bars indicate standard deviations (*n* = 6). (C) Effects of FeCl_3_, FeSO_4_, and PTIO on NO release. Levels of free NO were monitored amperometrically in a solution to which 100 μM NO donor SP-NO was added at *t* = 0 min. After 15 min (red arrow), 100 μM FeCl_3_, 100 μM FeSO_4_, or 200 μM PTIO was added to the solution. The results are representative of at least three independent experiments. *, *P ≤ *0.05; **, *P ≤ *0.01; ***, *P ≤ *0.001 versus untreated control samples.

To determine whether NO induces biofilm dispersal through the inhibition of iron uptake systems, the impact of the addition of exogenous iron on NO-induced dispersal was explored. Biofilms were first treated with different concentrations of ferrous iron and NO simultaneously for 30 min. After treatment with 100 μM of the NO donor SP-NO alone for 30 min, approximately 90% of biofilms were dispersed (Fig. 2A). In contrast, when ferrous iron was added to biofilms at the same time as NO, the dispersal response was inhibited in an iron dose-dependent manner (Fig. 2A), with only 40% of the biofilms dispersed after 30 min in the presence of 100 μM FeSO_4_. These data also show that in the presence of iron alone, the biofilm biomass increased compared to that of biofilms that had not received iron. This suggested two possibilities, (i) that iron may interfere with NO sensing and the induction of dispersal, or (ii) that iron may affect the dispersal process further downstream in the regulatory cascade. To address this, biofilms were first exposed to NO alone for 15 min, which is sufficient for the induction of dispersal as shown in our previous work (19), before iron was added to the cultures. Even when iron was added 15 min after NO, the biofilm biomass was found to increase in the presence of iron, with 100 μM FeSO_4_ resulting in 4.9- and 4.7-fold increases after 15 and 30 min, respectively (Fig. 2B). This suggested that the effect of iron on dispersal was not dependent on its presence during NO release, NO sensing, or the onset of dispersal. Similar increases in biofilm biomass were found after cells were exposed to 100 μM ferric iron FeCl_3_ (Fig. 2B), indicating that this phenotype was not dependent on the iron oxidation state (i.e., ferrous Fe^2+^ versus ferric Fe^3+^).

Iron is known to have a direct effect on free NO via redox reactions (21). To investigate whether iron directly scavenges NO and consequently inhibits NO-induced dispersal, NO-specific electrodes were used to measure the amount of free NO released from NO donor SP-NO in the presence of FeCl_3_, FeSO_4_, or the NO scavenger PTIO (2-phenyl-4,4,5,5-tetramethylimidazoline-1-oxyl 3-oxide) (Fig. 2C). In the absence of any iron or scavenger, the amount of NO liberated from SP-NO reached a steady state of approximately 4 μM within 15 min. The subsequent addition of 100 μM FeSO_4_ caused a dramatic reduction of free NO within 5 min, after which, the amount of NO increased to similar levels as the control (no iron). In contrast, 100 μM FeCl_3_ had no effect on the amount of NO released. The exposure of P. aeruginosa to either form of iron resulted in increased biofilm biomass (Fig. 2D).

To further confirm that the inhibitory effect of iron on dispersal was not related to the scavenging of NO by iron, the NO scavenger PTIO was added to the SP-NO solution instead of iron. The addition of 200 μM PTIO caused a dramatic reduction of free NO that lasted for the duration of the experiment (Fig. 2C). PTIO was also added to cultures that had been dispersed by NO for 15 min. After 15- and 30-min exposures to PTIO, the biofilms remained dispersed (Fig. 2D). Thus, in contrast to that with iron addition, PTIO-mediated scavenging of NO did not lead to hyperbiofilm formation. Ferrous iron showed a transient reduction in NO, inhibited biofilm dispersal, and induced hyperbiofilm formation. Ferric iron did not scavenge NO but inhibited dispersal and induced hyperbiofilm formation. These results suggest that iron overrides the NO-induced dispersal response and that this effect is independent of NO scavenging. Subsequent experiments were performed with FeCl_3_ to avoid issues from the short-term loss of NO after adding FeSO_4_.

### Biofilms formed in the presence of iron can be dispersed by NO.

**FIG 3:**
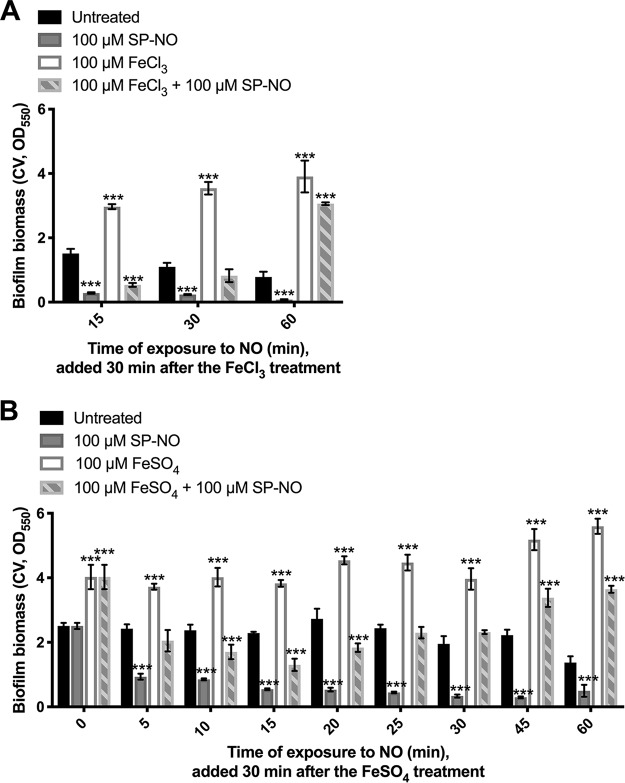
Biofilms formed in the presence of iron can be dispersed by NO. (A) P. aeruginosa biofilms were grown in multiwell plate batch cultures for 6 h and pretreated with 100 μM FeCl_3_ for 30 min. Biofilms were then treated with 100 μM SP-NO for 15, 30, or 60 min before CV staining. (B) Six-hour biofilms were pretreated with 100 μM FeSO_4_ for 30 min. Biofilms were then treated with 100 μM SP-NO for 5 to 60 min before CV staining. Error bars indicate standard deviations (*n* = 4). ***, *P ≤ *0.001 versus untreated control samples.

Since iron did not appear to inhibit dispersal by directly scavenging NO, an alternative possibility is that it may induce a cellular response that shuts down the ability of P. aeruginosa cells to disperse in the presence of NO signals. To explore whether the presence of iron can fully abolish NO-induced dispersal or whether iron and NO compete through the same regulatory pathway, the order of NO and iron addition was switched. P. aeruginosa cells were first exposed to iron for 30 min before NO treatment for 15 to 60 min. Biofilms that had not been treated with iron and biofilms that had been treated with 100 μM FeCl_3_ for 30 min were dispersed by 81% and 82%, respectively, after subsequent exposure to 100 μM SP-NO for 15 min (Fig. 3A). However, 60 min after the addition of NO, biofilms not pretreated with iron remained dispersed, while biofilms pretreated with 100 μM FeCl_3_ increased in biomass 3.9-fold compared to that of untreated control biofilms. Similar results were obtained when biofilms were pretreated with FeSO_4_ before NO (Fig. 3B). To better understand these changes in biofilm biomass, similar experiments were performed with shorter time frames. Surprisingly, biofilms pretreated with iron dispersed in the first 15 min of exposure to NO before increasing their biomass again. The biomass of biofilms pretreated with iron and subsequent exposed to NO decreased by 45%, 58%, and 66% compared to that of iron-pretreated biofilms after 5, 10, and 15 min, respectively. However, after 20 min, the biomass of these biofilms started to increase and, after 60 min, reached 2.7-fold of that of the untreated control biofilms. Overall, these results reveal that biofilms that are formed in the presence of iron can still be dispersed by NO and that the biofilm biomass increases rapidly in the presence of iron.

### Iron induces rapid (re)attachment of NO-induced dispersed cells and planktonic cells.

**FIG 4:**
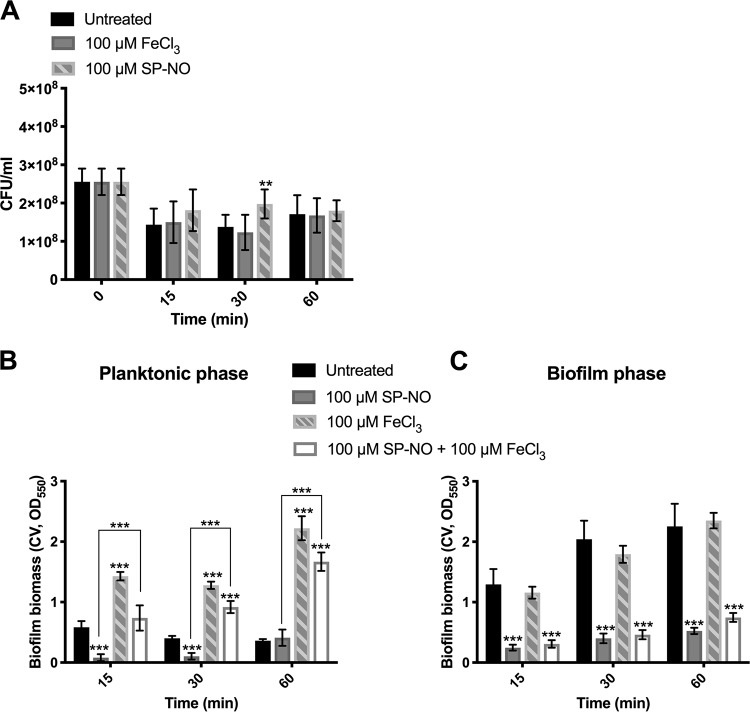
Iron overrides NO-induced biofilm dispersal mainly by promoting rapid attachment of suspended cells. (A) The effects of iron and NO on the growth of P. aeruginosa were tested. P. aeruginosa biofilms were grown in multiwell plate batch cultures for 6 h, and subsequently, supernatants containing planktonic cells were collected and transferred to 50-ml tubes. Bacterial cells were incubated in the absence and presence of 100 μM FeCl_3_ or SP-NO for 15, 30, and 60 min and CFU were calculated. Error bars indicate standard deviations (*n* = 9). Furthermore, P. aeruginosa biofilms grown in multiwell plate batch cultures for 6 h and subsequently dispersed by 100 μM NO donor for 15 min. Supernatants containing planktonic cells and dispersed cells were then transferred to a new empty multiwell plate, and fresh M9 medium was added to the original plate with the remaining biofilms. The two plates containing either the supernatants (B) or the remaining biofilms (C) were then incubated as before and treated or not with 100 μM FeCl_3_ for 15, 30, and 60 min before CV staining. Error bars indicate standard deviations (*n* = 6). **, *P* ≤ 0.01; ***, *P* ≤ 0.001 versus untreated control samples or between different samples.

In the above-mentioned multiwell plate batch culture biofilm dispersal assay, iron was added directly to 6-h bacterial cultures composed of biofilms and planktonic cells. It is possible that the increase in biofilm biomass observed in those experiments was due to an increased growth of the remaining biofilm or to the rapid attachment of the suspended cells containing both dispersed and planktonic cells. First, to determine if iron supplementation increases cell growth and if NO treatment inhibits cell growth, the CFU of P. aeruginosa cells that had been previously incubated in multiwell plate batch cultures for 6 h and subsequently exposed to iron and NO was enumerated. After adding iron for 15, 30, or 60 min, the CFU did not increase (Fig. 4A), suggesting that iron does not promote cell growth under these conditions. Furthermore, after treatment with NO for 15, 30, or 60 min, the CFU did not decrease, indicating that NO was nontoxic at the concentrations used in this study (Fig. 4A).

Second, the planktonic phase and the biofilm phase of pregrown bacterial cultures were separated after NO treatment and before adding iron in order to distinguish a potential effect of iron on the attachment of suspended cells from that of increased growth of already attached biomass. Culture supernatants were transferred to another well, and fresh medium was added to the remaining biofilms. Iron was then added to the wells containing only the suspended cells or the wells containing only the nondispersed biofilms. The data show that, in the absence of NO, the suspended cells attached rapidly when put in contact with a clean uncolonized surface. Furthermore, the attached biomass increased by 2.4-, 3.2-, and 6.2-fold after adding 100 μM FeCl_3_ for 15, 30, and 60 min, respectively (Fig. 4B). In the presence of NO (without iron), the suspended cells showed very little attached biomass after being transferred to, and incubated in, a clean new plate for 15 and 30 min. The biomass of NO-treated suspended cells increased by 9.1-, 8.8-, and 4.1-fold in the presence of FeCl_3_ for 15, 30, and 60 min, respectively (Fig. 4B). Those results suggest that NO prevents the rapid attachment of planktonic cells, while iron induces rapid attachment regardless of the presence of NO. In contrast, the dispersing effect of NO lasted for 60 min, and the presence of iron did not enhance the biomass of the NO-treated remaining biofilms (Fig. 4C). Overall, the above-mentioned results indicate that iron increases the biofilm biomass of NO-treated biofilms mainly by promoting the rapid attachment of planktonic cells or the reattachment of dispersed cells rather than by accelerating the growth of cells already within the remaining biofilms.

We repeated the experiment shown in Fig. 4B and C using a clinical isolate P. aeruginosa PA_D25 (42), which was collected from a patient with ventilator-associated pneumonia. As shown in Fig. S1, although the clinical strain was a poor biofilm former overall, the biofilms were dispersed by NO, and iron increased the biofilm biomass of NO-treated biofilms. As observed for strain PAO1, this effect of increased biomass was mainly through the promotion of rapid attachment of planktonic cells or the reattachment of dispersed cells (Fig. S1A) rather than through the acceleration of the growth of the remaining biofilms (Fig. S1B).

### *psl* is required for iron-induced fast attachment of planktonic cells.

**FIG 5:**
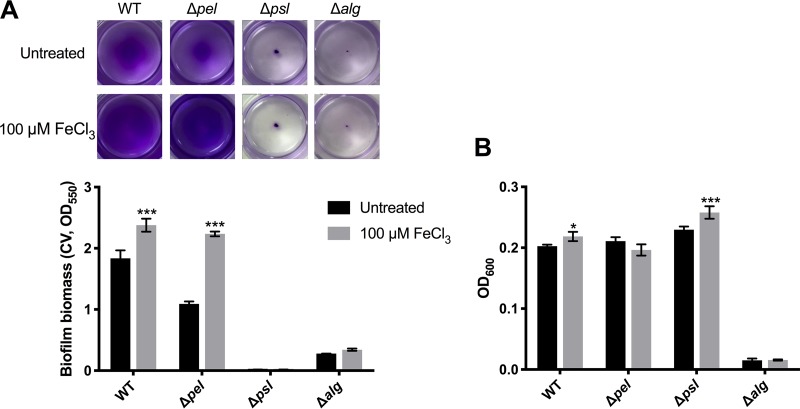
Iron does not promote rapid attachment of planktonic cells of P. aeruginosa Δ*psl* mutant. Biofilms of P. aeruginosa wild-type (WT) and Δ*pel* (isogenic *pelF* deletion mutant), Δ*psl* (isogenic *pslA* deletion mutant), and Δ*alg* (isogenic *alg8* deletion mutant) mutant strains were grown in multiwell plate batch cultures for 6 h and treated with 100 μM FeCl_3_ for 30 min before CV staining of the biofilm biomass (A) and OD_600_ measurement of the planktonic biomass (B). Each image represents the stained biofilms. Error bars indicate standard deviations (*n* = 3). *, *P* ≤ 0.05; ***, *P* ≤ 0.001 versus untreated control samples.

Psl, Pel, and alginate are the three main exopolysaccharides involved in biofilm development and antibiotic resistance in P. aeruginosa. High levels of iron (50 and 100 μM) were recently reported to promote biofilm formation in P. aeruginosa by increasing the production of Psl (24). To determine if iron facilitated the rapid attachment of planktonic cells through inducing the biosynthesis of those exopolysaccharides, P. aeruginosa polysaccharide-deficient mutants, including Δ*pel*, Δ*psl*, and Δ*alg* strains, were tested (Fig. 5A). P. aeruginosa wild-type and Δ*pel* strains showed approximately 1.3- and 2.0-fold increases, respectively, in biofilm biomass after treatment with 100 μM FeCl_3_. The Δ*alg* mutant did not grow well under these experimental conditions and its optical density at 600 nm (OD_600_) remained at the detection limit, 0.01, after 6 h of incubation (Fig. 5B). In contrast, the Δ*psl* mutant grew well in the planktonic phase (Fig. 5B) but failed to form biofilms (Fig. 5A), suggesting that Psl is important for biofilm formation under these experimental conditions. Furthermore, no attachment of the Δ*psl* planktonic cells was found in the presence of 100 μM FeCl_3_ (Fig. 5A). The results presented above suggest that Psl is required for the iron-induced rapid attachment of planktonic cells, although it remains to be determined if iron induced the production of Psl to enhance attachment.

**FIG 6:**
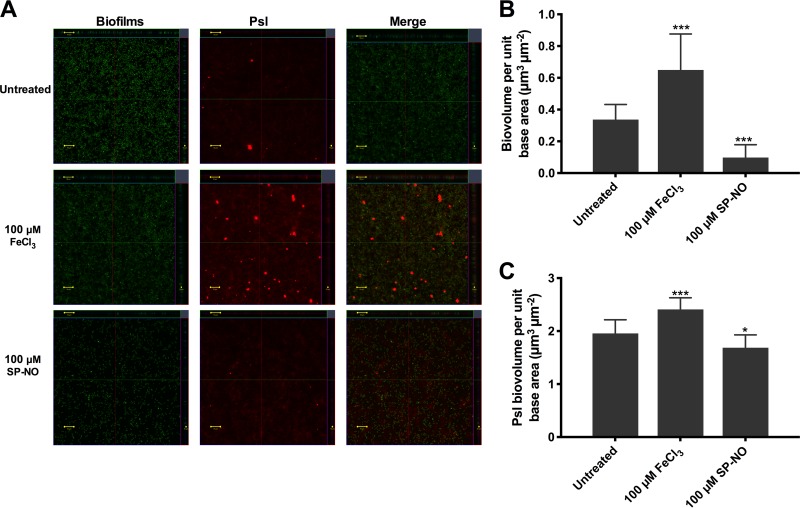
Psl production increased in iron-treated biofilms and slightly decreased after NO treatment. P. aeruginosa biofilms grown in multiwell plate batch cultures for 6 h and subsequently treated with or without 100 μM FeCl_3_ or 100 μM NO donor SP-NO for 30 min were stained with Syto9 and TRITC-HHA and analyzed by confocal microscopy (A). Biofilm cells appear green and Psl polysaccharides appear red. The main central images show horizontal (*x-y*) optical sections of the biofilms, and the side and top panels show vertical optical sections (*x-z* and *y-z*, respectively). Scale bars,10 μm. Image analysis was used to quantify biofilm (B) and Psl (C) signals from reconstructed 3-dimensional image stacks. Error bars indicate standard deviations (*n* = 12). *, *P* ≤ 0.05; ***, *P* ≤ 0.001 versus untreated control samples.

To investigate whether iron and NO influence biofilm development by controlling Psl production or other mechanisms, Psl of P. aeruginosa biofilms before and after iron or NO treatment were quantified by using a Psl-specific fluorescent stain, tetramethyl rhodamine isothiocyanate-labeled hippeastrum hybrid lectin (TRITC-HHA) (43), and microscopy analysis. After adding 100 μM FeCl_3_ for 30 min, biofilm biomass increased by 1.9-fold (Fig. 6B) and Psl production increased by 1.2-fold (Fig. 6C). These results confirmed that 100 μM FeCl_3_ promoted Psl production and induced cell attachment. In contrast, treatment with 100 μM NO donor for 30 min resulted in 71% biofilm dispersal (Fig. 6B) but only led to a slight reduction (14%) in the Psl-bound TRITC-HHA fluorescent signals (Fig. 6C). Confocal images showed that most Psl remained on the surface of the microtiter wells after NO treatment (Fig. 6A). Overall, these results suggest that iron induces attachment by promoting the production of Psl, while NO may induce dispersal not only by degrading Psl but also by altering another mechanism of P. aeruginosa attachment (discussed below).

### Iron-limited biofilms become more sensitive to NO.

**FIG 7:**
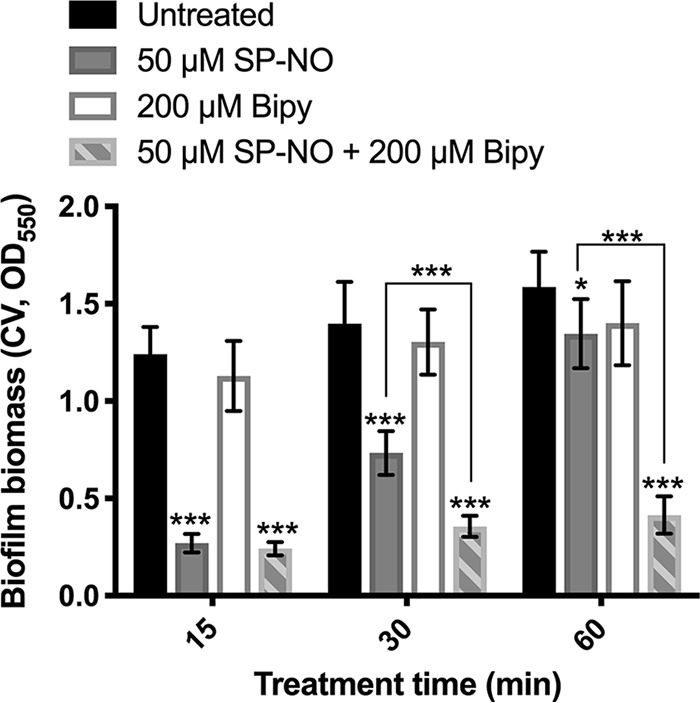
Iron-limited biofilms become more sensitive to NO. Biofilms grown in multiwell plate batch cultures for 6 h were treated with 50 μM SP-NO, 200 μM Bipy, or the combination of both for 15, 30, and 60 min. Biofilm biomass was quantified by CV staining. Error bars indicate standard deviations (*n* = 6). *, *P* ≤ 0.05; ***, *P* ≤ 0.001 versus untreated control samples or between different samples.

The above-presented data show that NO induced a concomitant decrease in pyoverdine production at the same time as inducing the dispersal of biofilm cells and that freshly dispersed cells rapidly reattach in the presence of iron. These observations suggest that depleting iron by the use of a chelator could potentially inhibit the reattachment of dispersed cells and enhance the dispersal effect of NO. To investigate whether iron depletion enhanced or interfered with NO-induced dispersal, biofilms were treated with NO in the presence or absence of the iron chelator, 2,2′-bipyridine (Bipy). The iron chelator alone had no significant impact on biofilm biomass (Fig. 7). In these experiments, the NO donor SP-NO was used at 50 μM, a lower concentration that results in the rapid depletion of NO and reattachment of biofilms, with the biomass of SP–NO-treated biofilms increasing to 53% of the untreated biofilm biomass after 30 min and to 85% after 60 min. In contrast, when 50 μM NO donor was added together with 200 μM Bipy, 74% of the biofilms were removed after 30 min, and this effect was prolonged after 60 min (Fig. 7). Taken together, these results indicate that iron depletion potentiates NO-induced biofilm dispersal.

## DISCUSSION

### Interplay between iron and NO in controlling biofilm development.

NO and iron are two important environmental cues that control biofilm formation. This study explored the interplay between these signaling molecules in the regulation of P. aeruginosa biofilms and found that (i) NO induces a decrease in several iron acquisition-related genes as well as a decrease in pyoverdine production, (ii) iron overrides NO-induced biofilm dispersal by promoting a rapid reattachment of dispersed cells, which involves Psl production, and (iii) combined treatments of NO with an iron chelator enhance biofilm dispersal.

A link between NO-mediated dispersal and the reduced expression of iron acquisition-related genes has been observed before (7, 27). However, in these studies, the NO donor that was used, sodium nitroprusside, contains an iron moiety and was previously found to release iron ions, which could have a potential impact on pyoverdine production independent of NO (44). Here, the effects were observed using a NONOate NO donor that does not contain iron. Bacteria in biofilms are usually associated with a physiology indicative of iron limitation, which is likely due to the high cell density and limited availability for this nutrient. The potential coregulation of dispersal with reduced expression of iron acquisition is interesting, since dispersed cells are likely to encounter increased levels of iron once they are treated with NO. It is unclear why *bfrB* is highly expressed after NO treatment. One possibility is that NO induces *bfrB* through Fur, since *bfrB* is predicted to be regulated by Fur (45, 46). A recent study revealed that accumulation of iron binding by BfrB induces acute iron deprivation in the cytoplasmic space as well as the derepression of iron acquisition genes (41). However, in our study, NO was found to induce *bfrB* expression, while iron acquisition genes were repressed. Our results may differ from those in the previous study in that while 15 min after NO treatment, *bfrB* is highly induced, the binding of intracellular iron by BfrB may not be significant, thus maintaining the repression of iron acquisition genes. We also tested a *bfrB* mutant and observed that it still dispersed to the same extent as the P. aeruginosa PAO1 wild type (data not shown), suggesting that BfrB does not contribute to biofilm dispersal. Compared to the strong repression of several pyoverdine synthesis genes, the reduction of pyoverdine by NO was much less. It is possible that NO does not affect the existing pool of pyoverdine at 6 h but rather inhibits transcriptional control; hence, changes in pyoverdine levels may lag well behind the repression of gene expression. Although the exact mechanism by which NO regulates *pvd* genes and pyoverdine production remains to be elucidated, this effect is likely to involve c-di-GMP. Indeed, NO is known to induce a reduction in c-di-GMP levels (7), and in turn, decreased c-di-GMP has been found to abolish pyoverdine production (29). The results of this study showed that the addition of iron, which is also known to reduce *pvd* expression, enhanced the attachment of bacteria rather than promoting dispersal, which suggests that the decrease of *pvd* induced by NO was unlikely to be the cause of the downstream dispersal response but may rather be a parallel unrelated regulatory effect.

### NO- and iron-induced signaling pathways appear to be independent but converge on the downstream attachment effectors.

The results shown here suggest that NO and iron do not operate via overlapping signaling pathways, as iron does not strictly inhibit the dispersal response and NO-dispersed cells can still attach in response to iron. However, the responses to both iron and NO appear to involve the exopolysaccharide Psl. A recent study found that high iron (50 and 100 μM FeCl_3_) promotes Psl production and thus induces biofilm formation (24), which correlates with the data presented here. Fast attachment responses involving Psl polysaccharides have already been observed in P. aeruginosa, including in response to surfactant stress. For example, SDS surfactants have been shown to induce aggregation and attachment of suspended cells within 45 min via c-di-GMP and Psl (47). Interestingly, while the addition of iron can induce attachment in a Psl-dependent manner, the results presented here suggest that chelation of iron alone in the absence of NO treatment does not promote dispersal (Fig. 7). In P. aeruginosa, the intracellular concentration of c-di-GMP is known to regulate Psl production at both the transcriptional and translational levels (44). While it has been shown, as indicated above, that NO exposure results in reduced c-di-GMP concentrations, it remains unclear if there is a direct link between NO and Psl production. In contrast, the effect of iron on Psl does not appear to involve c-di-GMP, as it was previously found that the addition of iron had no impact on c-di-GMP levels (24). Another important regulatory pathway that drives the transition between biofilms and planktonic cells in P. aeruginosa is the acylated homoserine lactone (AHL)-based QS system. High levels of iron have been suggested to promote Psl production through the repression of the QS-controlled genes *rhlAB*, *rhlI*, and *rhlR*, thus reducing the synthesis of rhamnolipids as well as inhibiting *amrZ*, which encodes a transcriptional factor that inhibits transcription of the *psl* operon (48–50). Therefore, it is possible that iron controls Psl by modulating QS. A potential link between iron, biofilms, and QS could be explored in future studies by investigating QS mutants using the iron-induced fast attachment assay reported here.

Moreover, in this study, NO had little impact on Psl levels, and most Psl remained attached to the surface after NO treatment, despite dispersal of most of the biofilm (Fig. 6). Intriguingly, the dispersal response to NO has been found to require the periplasmic protease LapG (17), which can cleave the protein adhesin CdrA off the cell surface (51). CdrA is known to either cross-link Psl polysaccharide polymers and/or tether the cells to the Psl polysaccharides (3). Taken together, these observations suggest that the addition of NO causes the cleavage of CdrA from the cell surface via c-di-GMP and LapG and therefore breaks the link between biofilm cells and Psl, finally resulting in the release and dispersal of bacteria.

### Combined treatments of NO and iron chelators may improve biofilm control.

In natural environments, bacteria can encounter various levels of available iron. For example, iron is present in wastewater at an average concentration of 9 μM (52), while in the cystic fibrosis (CF) lung, the iron concentration varies from 2 to 130 μM (53–57). Therefore, the presence of iron could significantly impact the efficacy of NO when applied in industrial and clinical settings. A range of iron-chelating compounds for biofilm control has been studied previously (58–60). In this work, the iron chelator Bipy was used in combination with NO, and it was observed that NO-treated biofilms were more sensitive to iron limitation and dispersed to a greater degree than biofilms in the absence of iron chelator. Furthermore, previous studies have also shown that the combination of NO, iron chelator, and tobramycin efficiently reduces the survival of P. aeruginosa dispersed cells (27). Therefore, simultaneous treatments of biofilms with NO, an iron chelator, and biocides could be a powerful way to remove and kill biofilms in iron-rich environments.

In summary, this study has shown that two important environmental signals, iron and NO, control biofilm development in opposing ways through different pathways, which appear to be both linked to the polysaccharide Psl. Furthermore, iron chelator and NO were found to have a synergetic effect in dispersing biofilms, which suggests new ways for improving the use of NO in biofilm control strategies.

## MATERIALS AND METHODS

### Bacteria and growth conditions.

The P. aeruginosa PAO1 wild type (WT) strain (61) and P. aeruginosa Δ*pel* (isogenic *pelF* deletion mutant [62]), Δ*psl* (isogenic *pslA* deletion mutant [62]), and Δ*alg* (isogenic *alg8* deletion mutant [62]) mutant strains were used. Bacteria were routinely grown in Luria-Bertani (LB) Miller broth (BD Difco) for 16 h at 37°C with shaking at 200 rpm to prepare cells for experiments.

### Biofilm dispersal and attachment assays.

Biofilms were grown and dispersed as previously described (63) with some modifications, as explained in our previous work (19). For bacterial attachment assays, P. aeruginosa cultures were grown in multiwell plates using the same experimental settings as for biofilm dispersal assays. After 6 h, FeCl_3_ (Sigma-Aldrich), FeSO_4_ (Sigma-Aldrich), or the NO scavenging compound 2-(4-carboxyphenyl)-4,4,5,5-tetramethylimidazoline-1-oxyl-3-oxide potassium salt (carboxy-PTIO potassium salt; Sigma-Aldrich) was added to the cultures that had or had not received NO treatment. The final concentration of iron salts was 100 μM and the concentration of PTIO was 200 μM. The plates were incubated for a further 5 min to 60 min. After the final incubation, biofilm biomass was quantified using crystal violet (CV) staining as described previously (19).

### Pyoverdine quantification.

The production of pyoverdine in P. aeruginosa cultures was quantified by measuring the natural fluorescence of the culture supernatants (excitation wavelength, 400 nm; emission wavelength, 450 nm) (Infinite Pro2000 microplate reader; Tecan) (64), and the pyoverdine production level was normalized to the OD_600_ values for each well.

### Amperometric measurements of NO.

The concentration of NO liberated from NO donor was measured amperometrically by using a TBR1025 free radical analyzer (World Precision Instruments) equipped with an NO specific ISO-NOP 2-mm electrode, with a detection range from 1 nM to 100 μM and calibrated by using methylamine hexamethylene methylamine (MAHMA) NONOate (Cayman Chemical) as the NO donor. After allowing the amperometric signal to stabilize in M9 salts, 100 μM SP-NO was added. Then, after 15 min, 100 μM FeSO_4_, 100 μM FeCl_3_, or 200 μM PTIO was added. These experiments were repeated at least 3 times.

### Biofilm, Psl staining, and microscopy analysis.

P. aeruginosa wild-type biofilms grown in multiwell plate batch cultures for 6 h and subsequently left untreated or treated with 100 μM FeCl_3_ or 100 μM NO donor SP-NO for 30 min were rinsed once with phosphate-buffered saline (PBS) before being stained with 50 μg/ml tetramethyl rhodamine isothiocyanate-labeled hippeastrum hybrid lectin (amaryllis) (TRITC-HHA; EY Labs, Inc.) for 1 h, as previously described (43). Biofilms were subsequently rinsed twice with PBS before being stained with Syto9 (Molecular Probes, Inc.). Briefly, 1.5 μl Syto9 was diluted in 1 ml of PBS, and then 0.5 ml of this solution was added to each well and incubated at room temperature in the dark for 15 min. Images of untreated and iron- or NO-treated biofilms were acquired through the bottom surfaces of the culture wells by using inverted confocal laser scanning microscopy (CLSM) (LSM 780; Carl Zeiss Microscopy). Biofilm quantification was performed using the IMARIS software package (Bitplane AG).

### Statistical analysis.

Multivariate analyses were performed using one-way analyses of variance (ANOVAs) and two-way ANOVAs, followed by Sidak’s posttests for individual comparisons.

## Supplementary Material

Supplemental file 1
