# Supplementary material for: Nitric Oxide and Iron Signaling Cues Have Opposing Effects on Biofilm Development in Pseudomonas aeruginosa
Source: Appl Environ Microbiol. 2019 Jan 23;85(3):e02175-18. doi: 10.1128/AEM.02175-18 (PMC6344620; doi:10.1128/AEM.02175-18)
Supplement: Supplemental file 1 [file ec98dc65d1bfa02b90f1ef97327104f3_AEM.02175-18-s0001.pdf]

SUPPLEMENTAL MATERIAL

Text S1. Materials and Methods

Biofilm cultures before nucleotide extraction

Biofilms were grown as previously described (1) with some modifications (2). Briefly, *P. aeruginosa* PAO1 bacteria cultures were incubated at 37°C with shaking at 180 rpm for 6 h or 5.75 h. After incubation, untreated planktonic (supernatant) cells and biofilm cells were collected from plates that were incubated for 6 h. For plates that were incubated for 5.75 h, biofilms were washed with phosphate-buffered saline (PBS; containing 137 mM NaCl, 2.7 mM KCl, 2 mM KH_2PO_4 and 10 mM Na_2HPO_4, pH 7.4) and were treated with fresh M9 minimal medium (containing 9 mM NaCl, 22 mM KH_2PO_4, 48 mM Na_2HPO_4, 19 mM NH_4Cl, 2 mM $MgSO_4$, 100 μM $CaCl_2$ and 0.4% glucose, pH 7.0) in the presence of 100 μM NO donor spermine NONOate (SP-NO) (Cayman Chemical) for 15 min. Subsequently, NO treated non-dispersed, remaining biofilm cells and dispersed cells were collected. Untreated biofilms and remaining biofilms were collected from the bottom surface of each well using a sterile cell scraper and immediately suspended in RNAprotect reagent (Qiagen). After collection, all samples were centrifuged at 5, 000 × *g* for 10 min and suspended in 1 ml RNAprotect reagent (Qiagen) in Eppendorf tubes. Tubes with suspension were vortexed for 15 s and incubated at room temperature for 5 min before centrifuged at 5, 000 × *g* for 10 min. Finally, the supernatants in each tube were discarded and the cell pellets were used for RNA extraction. This experiment was performed in triplicate.

Total RNA preparation, sequencing and data analysis

Total RNA was extracted from all samples using a Direct-zol™ RNA MiniPrep kit (Zymo Research) as per the manufacturer's protocol. DNase treatment was conducted using the TURBO

DNA-*free* Kit (Ambion) as per the manufacturer's protocol. RNA was concentrated and purified using the RNA Clean & Concentrator™-5 kit (Zymo Research) as per the manufacturer's protocol. The integrity and concentration of the total RNA was assessed with an Agilent 2100 Bioanalyzer (Agilent Technologies) and Qubit 2.0 Fluorometer (Invitrogen). RNA library preparation was performed according to the TruSeq Stranded mRNA protocol (Illumina) without the oligo-dT mRNA purification step and the libraries were sequenced using the HiSeq2500 sequencer (Illumina). mRNA sequencing data was analysed using the TopHat and Cufflinks software programs (3). The sequence reads were mapped onto the *P. aeruginosa* PAO1 reference genome (downloaded from NCBI [NC_002516.2]).

Table S1. Genes that were changed in NO treated remaining biofilms compared to untreated biofilms in *Pseudomonas aeruginosa* (with a P-value cut-off of 0.05 and a fold change cut-off of 4)

Gene	Fold change	COG
bfrB	71.8	Inorganic ion transport and metabolism
femI	-25.2	Transcription
tonB1	-11.4	Cell envelope biogenesis, outer membrane
phuS	-11.1	Inorganic ion transport and metabolism
PA4156	-9.5	Inorganic ion transport and metabolism
pvdQ	-9.1	General function prediction only
phzC1	-9.0	Amino acid transport and metabolism
phzD1	-9.0	Secondary metabolites biosynthesis, transport, and catabolism
phzE1	-9.0	Amino acid transport and metabolism / Coenzyme metabolism
phzA1	-8.0	Not available
phzC2	-7.6	Amino acid transport and metabolism
phzD2	-7.6	Secondary metabolites biosynthesis, transport, and catabolism
phzE2	-7.6	Amino acid transport and metabolism / Coenzyme metabolism
fpvA	-7.6	Inorganic ion transport and metabolism
pvdA	-7.3	Secondary metabolites biosynthesis, transport, and catabolism
phuV	-7.2	Inorganic ion transport and metabolism
hasR	-6.9	Inorganic ion transport and metabolism
PA1302	-6.8	Inorganic ion transport and metabolism
pvdN	-5.8	Posttranslational modification, protein turnover, chaperones
phzG1	-5.6	Coenzyme metabolism
phzB2	-5.5	Not available
pvdO	-5.1	Function unknown
phzG2	-5.1	Coenzyme metabolism
pchA	-5.1	Coenzyme metabolism / Secondary metabolites biosynthesis, transport, and catabolism
pchB	-5.1	Amino acid transport and metabolism
pchC	-5.1	Secondary metabolites biosynthesis, transport, and catabolism
pchD	-5.1	Secondary metabolites biosynthesis, transport, and catabolism

fpvB	-5.0	Inorganic ion transport and metabolism
pchR	-4.8	Transcription
phzF1	-4.6	General function prediction only
optI	-4.5	Inorganic ion transport and metabolism
PA2407	-4.5	Inorganic ion transport and metabolism
PA2408	-4.5	Inorganic ion transport and metabolism
PA2409	-4.5	Inorganic ion transport and metabolism
PA2410	-4.5	Inorganic ion transport and metabolism
PA3410	-4.5	Transcription
phzF2	-4.3	General function prediction only

Table S2. Genes that were changed in NO dispersed cells compared to untreated planktonic cells in *P. aeruginosa* (with a P-value cut-off of 0.05 and a fold change cut-off of 4)

Gene	Fold change	COG
bfrB	77.0	Inorganic ion transport and metabolism
phzA1	-7.0	Not available
pvdA	-5.6	Secondary metabolites biosynthesis, transport, and catabolism
phzB1	-5.3	Not available
pvdS	-5.3	Transcription
PA5217	-4.5	Inorganic ion transport and metabolism
foxI	-4.1	Transcription

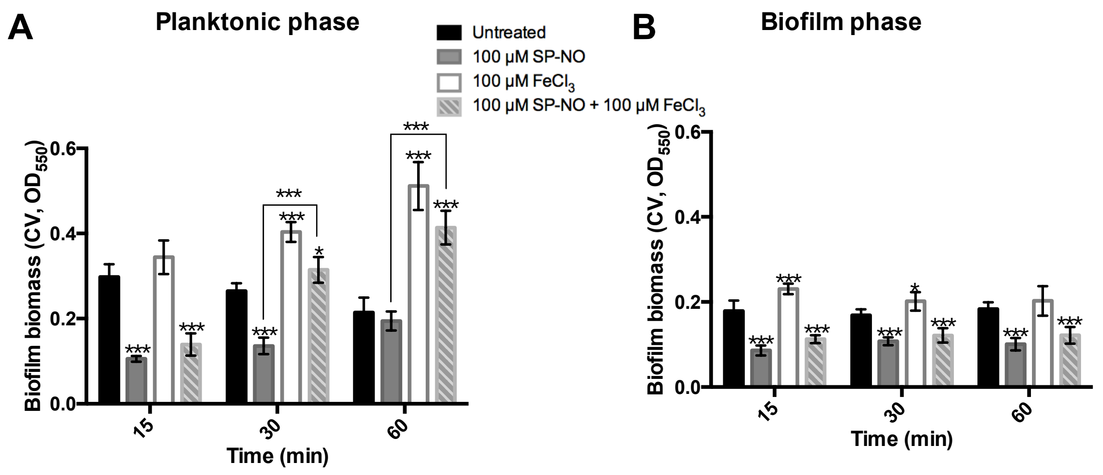

FIG S1. Iron overrides NO induced biofilm dispersal mainly via promoting rapid attachment of suspended cells. Biofilms of the *P. aeruginosa* PA_D25 strain grown in multiwell plate batch cultures for 6 h and subsequently dispersed by 100 µM NO donor for 15 min. Supernatants containing planktonic cells and dispersed cells were then transferred to a new empty multiwell plate and fresh M9 medium was added into the original plate with the remaining biofilms. The two plates containing either the suspended, planktonic cells (A) or the remaining biofilms (B) were then incubated as before and treated or not with 100 µM $FeCl_3$ for 15, 30 and 60 min before CV staining. Error bars indicate standard deviation (n = 6). Asterisks indicate statistically significant differences compared to untreated control samples or between different samples ($P \leq 0.05$).

REFERENCES

1. **Barraud N, Moscoso JA, Ghigo JM, Filloux A.** 2014. Methods for studying biofilm dispersal in *Pseudomonas aeruginosa*. Methods Mol Biol **1149:**643-651.
2. **Zhu X, Oh HS, Ng YCB, Tang PYP, Barraud N, Rice SA.** 2018. Nitric oxide-mediated induction of dispersal in *Pseudomonas aeruginosa* biofilms is inhibited by flavohemoglobin production and is enhanced by imidazole. Antimicrob Agents Chemother **62:**e01832-01817.
3. **Trapnell C, Roberts A, Goff L, Pertea G, Kim D, Kelley DR, Pimentel H, Salzberg SL, Rinn JL, Pachter L.** 2012. Differential gene and transcript expression analysis of RNA-seq experiments with TopHat and Cufflinks. Nat Protoc **7:**562-578.
